# Painful Neuroma of the Skin

**Published:** 1881-12

**Authors:** 


### PAINFUL NEUROMA OF THE SKIN.

Dr. Louis A. Duhring gives, in the October number of the *American Journal of the Med. Sciences*, the post-mortem report of a case of this rare affection, the clinical history of which was originally reported by him in 1873, in the same journal. An illustration shows the brachial plexus which had been divided at the former operation, but the cut ends had re-united by means of a dense mass of cicatricial fibrous tissue. The disease was ascertained to be a skin disease strictly speaking. No other structure was in any way involved. A representation of the microscopical appearance of one of the tumors also appears, as an illustration to this article, which shows the disease to be that technically known as neuroma myelinicum of Virchow. The case therefore was one of true neuroma of the skin, one of the rarest diseases.

